# Preventive Health Checkup: Utilization, Motivators, and Barriers Among the General Population in a Rural District in Tamil Nadu, India

**DOI:** 10.7759/cureus.52529

**Published:** 2024-01-18

**Authors:** Gowrisankar Arulselvan, Senthilkumaran Chidambaram, Neethu George, Shagirunisha Rizvana, Pooja Narayan, Pooja Annamalai, Prathap Hariharan R Vadakaraiyan, Nirmala Rajagopal, Rock B Dharmaraj, Tamilarasan M

**Affiliations:** 1 Medicine, Dhanalakshmi Srinivasan Medical College and Hospital, Perambalur, IND; 2 Psychiatry, Government Theni Medical College, Theni, IND; 3 Community Medicine, Dhanalakshmi Srinivasan Medical College and Hospital, Perambalur, IND

**Keywords:** general population, prevention, preventive health check-up, motivators, barriers

## Abstract

Background: The main goals of preventive health care include the early detection of disease by screening, identifying and reducing disease risk factors, and improving the current disease processes. Moreover, early disease discovery in the latent stage facilitates prompt intervention, reducing morbidity and death.

Objective: To estimate the proportion of people who have availed preventive health check-ups in Perambalur and to determine the motivators and barriers to health check-ups among adults (age group: 18 years and above).

Materials and methods: We conducted a cross-sectional study among the general population from September 2021 to November 2021. A total of 436 participants were included in the study. A structured questionnaire was used to collect the socio-demographic characteristics and preventive health check-up details, along with the motivators and barriers. The data were entered in Microsoft Excel (Microsoft, Redmond, WA), and analysis was done using SPSS version 26 (IBM Corp., Armonk, NY).

Result: The mean ± SD of the age of the general population was 52.27 ± 21.09. Out of 436 participants, only 130 (29.82%) had undergone preventive health check-ups in the past. The subjects with young age (p = 0.006), those who obtained COVID-19 vaccination (p = 0.001), subjects with stable occupation (p = 0.002), and those with higher education (p < 0.001) (chi-square test) had preventive health check-ups. A significant association was found between motivators and barriers for age, gender, education, occupation, marital status, the presence of comorbidities, and vaccination status against COVID-19.

Conclusion: Preventive health check-up is still not up the ladder of health care for the general population. The health programmes should orient the public towards the concept of individual responsibility in health.

## Introduction

According to the World Health Organization, non-communicable diseases like diabetes and cardiovascular disease, which are brought on by a "combination of genetic, physiological, environmental, and behavioural factors", claim the lives of 41 million people annually or 71% of all fatalities. Every year, about 5.8 million people in India pass away from non-communicable diseases (heart and lung diseases, stroke, cancer, and diabetes), which translates to one in four Indians being at risk of passing away from non-communicable diseases before the age of 70 years. The disability-adjusted life years have increased by 6.6%, 4.4%, 2.2%, and 0.9% for cardiovascular disease, chronic respiratory disease, diabetes, and breast cancer, respectively [1].

The primary emphasis of health organizations' efforts to reduce fatalities from non-communicable diseases is cigarette use, physical inactivity, unhealthy food, and problematic alcohol consumption [2]. Along with tackling the risk factors, another major step in combating non-communicable diseases is preventive health check-ups. Preventive health check-ups deal with the identification and minimization of disease risk factors, existing disease course improvement, and early disease detection through screening. This has become crucial since it allows people to know about their health issues well within time [3]. Also, early diagnosis of disease in its latent phase aids in prompt intervention, lowering morbidity and death [3,4]. To achieve optimal health for the nation, individuals must be fully engaged in taking care of their health, modifying risk behaviours, and implementing preventative actions to lower future demand for health care. The utility of the services by health programmes like the National Program for Prevention and Control of Non-communicable Disease, the National Programme for Health Care of the Elderly, and the National Tobacco Control Programme by the public is always questionable. In a country like India where mandatory health check-ups are missing or due to the absence of a proficient health system like that of Western countries, the public must push themselves to undergo these health check-ups.

Aside from the public advantage, using healthcare services for preventive purposes provides significant individual benefits. Preventative health care gives people confidence and knowledge about their health and the health of those they care for [5]. The result of a systematic review showed that participation in preventive health check-ups was facilitated by attitudes, including the desire to understand one's risk for cardiometabolic disease, a sense of personal accountability, and concerns for one's health. Younger age, smoking, less education, attitudes such as not wanting to worry about the results or being susceptible to them, and a generally unfavourable attitude towards health screenings or prevention were barriers. Additionally, practical concerns like information and accessibility to appointments may have an impact on participation [6,7]. So this study was done to assess the motivators and barriers of the public in carrying out preventive health check-ups. The objective of our study is to estimate the proportion of people who have availed of preventive health check-ups in Perambalur and to determine the motivators and barriers to health check-ups among adults (age group - 18 years and above).

## Materials and methods

Study setting

We conducted a cross-sectional study among the general population of the Perambalur district from September 2021 to January 2022.

Sample size

The sample size was estimated with a 95% confidence interval and 5% absolute precision by using the following formula: n = Z_α_^2 ^PQ/d^2^ [Z_α_ = 1.96, P = 32%, Q = (100-32 = 68), d = 5]. The proportion was taken from a previous study where 32% of people go for regular check-ups [8]. The sample size is estimated to be 334. With a 10% non-response rate, the final estimated sample size was 367. The study collected data from 436 subjects.

Sampling and study participants

The rural field practice area of a tertiary medical college hospital was used to collect the data. The investigators assumed a consecutive sampling method to obtain the subjects. The investigators interviewed the head of the family or their spouse from each household. If either of the people were not present during the time of the survey or if the house was locked or no one was there above the age of 18 years, we visited the same house on another day to collect data. Members who were not present in the house for more than two visits were excluded from the study. The data were collected for a period of three months.

Ethics committee approval and informed consent

Institution Ethics Committee approval (IECHS/IRCHS/NO/119-2021) and informed consent were obtained before the start of the study. The subjects were told about the details of the study questionnaire and the objective of the study in their native language (Tamil) after establishing rapport with the subject.

Study tools

The objective was assessed using a questionnaire prepared via the G Suite application (Google, Mountain View, CA) and data were collected through the interviewer method. The subjects were interviewed by the investigator for approximately 20 minutes. The questionnaire contains two parts: the first part, i.e., sociodemographic details, includes age, gender, occupation, education, family income, height, weight, comorbidities, and vaccination status for COVID-19 along with COVID-19 positivity. The second part includes preventive health check-up motivators (10 phrases) and barriers (10 phrases) and responses to be reported in a three-point Likert scale for positive questions (agree = 3, neutral = 2, and disagree = 1) and reversely coded for negative questions. The total score for preventive health checkup motivators and barriers is 10-30 in which the score is categorized for motivators as low (≤18), medium (19-22), and high (>22), and barriers for preventive health checkup as also categorized as low (≤19), medium (20-24), and high (>24). This categorization is based on the percentiles (>75th = high, 25th to 75th = average, and <25th = low). Also, the study identified the top barriers and motivators. For this, a score for each item was obtained by multiplying the weightage by the frequency of responses. For each question, the maximum possible score was 1308 (436 x 3). The internal consistency of the 20 questions was assessed by Cronbach’s alpha where it came to 0.78.

Data analysis

The data collected were entered in Microsoft Excel (Microsoft Corporation, Redmond, WA) and analysed using SPSS version 26 software (IBM Corp., Armonk, NY). The chi-square test was used to assess general characteristics and utilization of preventive health check-ups. Pearson’s correlation, independent t-test, and one-way ANOVA were used to assess general characteristics and motivators/barriers of preventive health check-ups. Multiple logistic regression and linear regression were performed to find the model, which predicts the barriers and motivators. The predictor variables were identified using a regression model where all variables with a significance of <0.05 in the primary analysis were taken.

## Results

Basic characteristics of the subjects

A total of 436 subjects’ data were collected from the general population aged ≥18 years. The mean ± SD of the age of the general population was 52.27 ± 21.09. The demographic characteristics are represented in Table 1.

**Table 1:** Basic characteristics and COVID-19 history of study participants (n = 436) * Subjects with any one comorbidity were considered as a "Yes". ^#^ Subjects who have received at least one dose of COVID-19 vaccine were considered as a "Yes".

Characteristics		Frequency (n)	Percent (%)
Sex	Male	214	49.1
	Female	222	50.9
Education	Professional degree/postgraduate	64	14.7
	Graduate	83	19
	Diploma/Intermediate	69	15.8
	High school	43	9.9
	Middle school	34	7.8
	Primary school	46	10.6
	No formal education	97	22.2
Occupation	Agriculture	31	7.1
	Business	53	12.2
	Daily wager	25	5.7
	Retired/pension	11	2.5
	Salaried	117	26.8
	Unemployed	199	45.7
Marital status	Married for less than 5 years	20	4.6
	Married for 5-10 years	38	8.7
	Married for more than 10 years	210	48.2
	Separated	1	0.2
	Divorce	3	0.7
	Unmarried	81	18.6
	Widow/widower	83	19
Comorbidities^*^	Yes	191	43.8
	No	245	56.2
Diagnosed with COVID-19	Yes	127	29.1
	No	309	70.9
Vaccinated against COVID-19^#^	Yes	281	64.4
	No	155	35.6
COVID-19 vaccine, n = 281	COVISHIELD	224	79.7
	COVAXIN	46	16.4
	MODERNA	3	1.1
	SPUTNIK V	8	2.8

The proportion of subjects who availed of preventive health check-ups with associated factors

**Figure 1:**
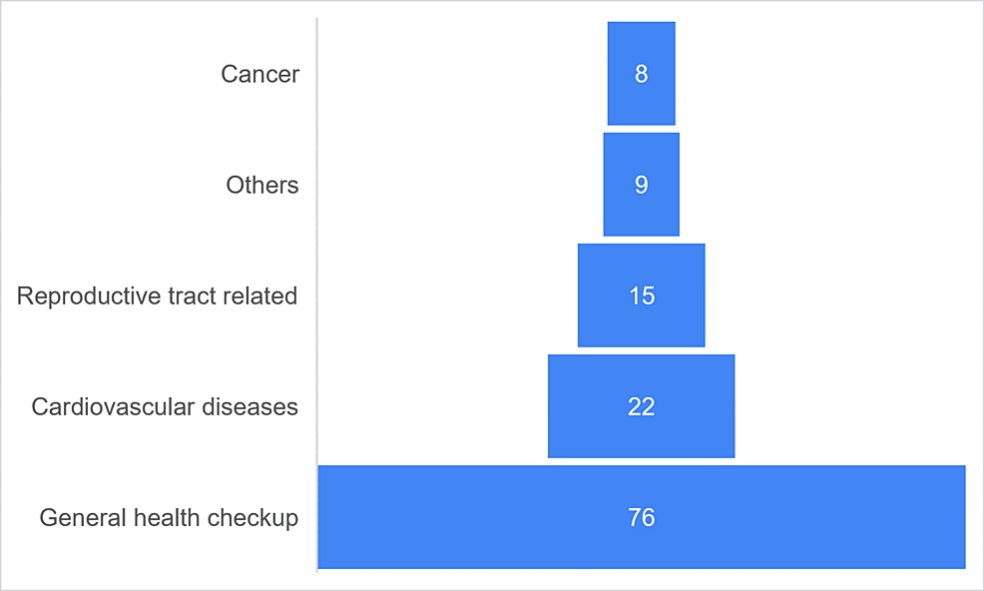
Condition in which preventive health check-up was done (n = 130) A funnel chart has been used to depict the conditions in which subjects availed for preventive health check-ups in the past one year. Others - check-ups done for eyes, teeth, bone density, etc.

Out of 436 participants, only 130 (29.82%) had undergone preventive health check-ups in the past. Figure 1 shows the conditions in which the subjects have availed of preventive check-ups in the past.

In the study, awareness regarding preventive health check-ups was acquired from health practitioners (79, 60.8%), friends/relatives (34, 26.2%), social networks (9, 6.9%), media (TV/radio) (5, 3.8%), and posters/banners (3, 2.3%). Further analysis in inferential statistics was done with the categorization of marital status as married/unmarried and others (single, widow, widower, divorced, and separated), education as nil/school/higher (degree, diploma, graduate, and postgraduate), and occupation as nil/stable (salaried, business, and retired with pension)/unstable (agriculture, daily wager).

The subjects with younger age (42.55 (18.88) vs. 56.40 (20.64)), COVID-19 vaccinated (38.1% vs. 14.8%), with stable occupation (38.1% vs. unstable (28.6%) vs. nil (22.6%)), and with higher education (41.7% vs. school education (30.1%) vs. nil education (3.1%)) (chi-square test, p-value < 0.01) availed of preventive health check-ups. The binary logistic regression model was performed to ascertain the effects of age, the status of COVID-19 vaccination, education, and occupation with availing preventive health check-ups (Hosmer-Lemeshow model of fit - chi-square value = 10.54, p-value = 0.23). The model was statistically significant where education came as significant in which subjects with higher education have 12.30 (95% CI: 3.22 to 46.97) times more chance to undergo a preventive health check-up.

Motivators/barriers in availing of preventive health check-ups

**Figure 2:**
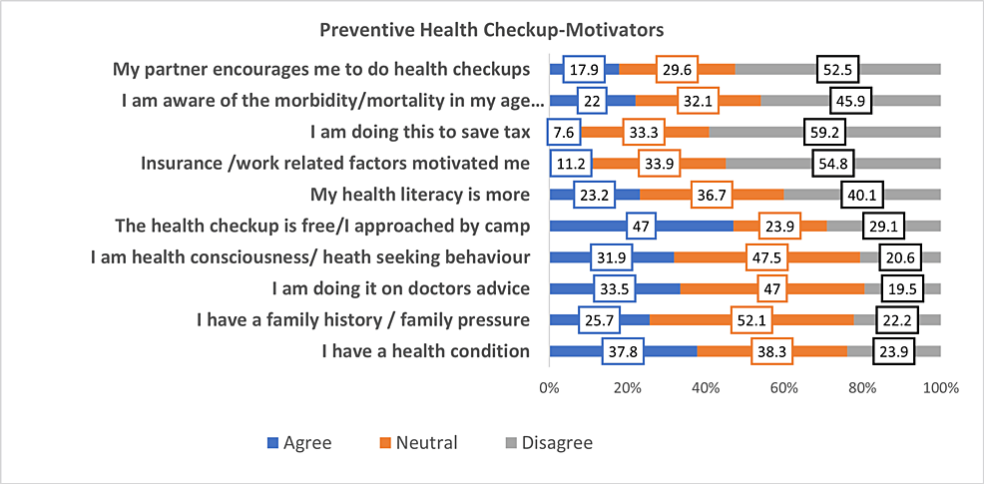
Response on motivators in availing of preventive health check-up Bar chart representing the responses on motivators, which are depicted as percentages.

**Figure 3:**
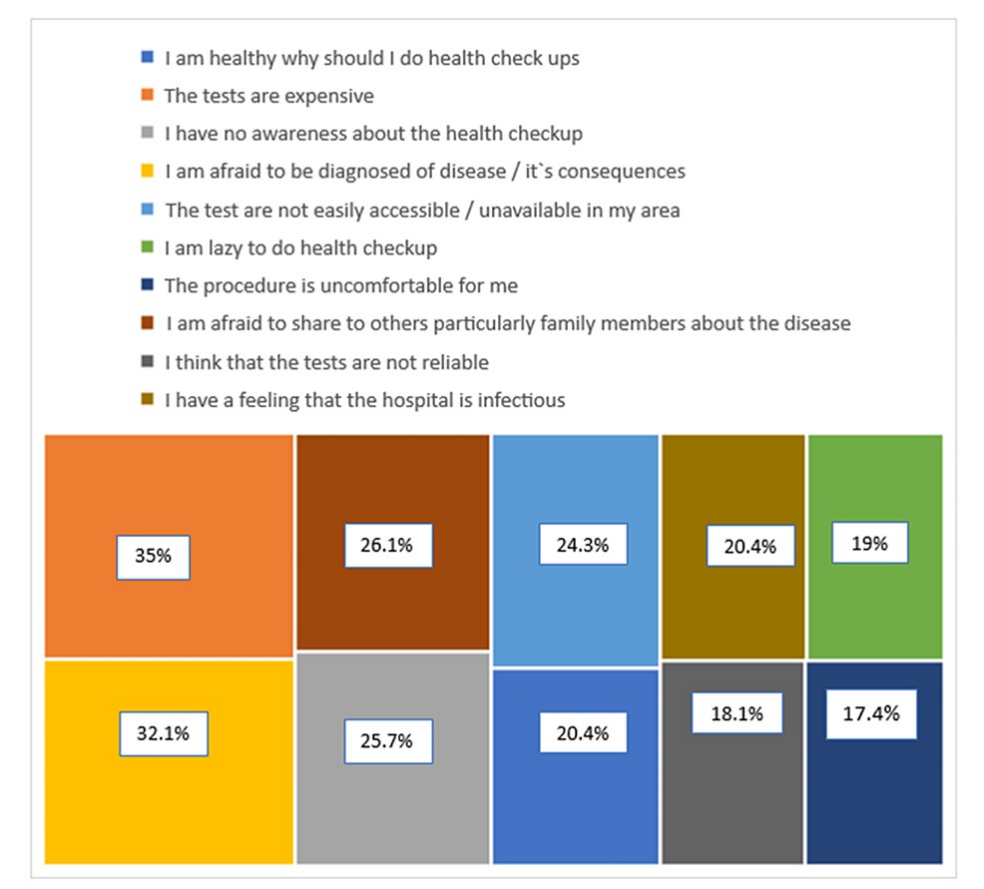
Responses on barriers to availing of preventive health check-up The treemap diagram is represented as rectangles in terms of the percentages of subjects who agreed to the subsequent response.

**Figure 4:**
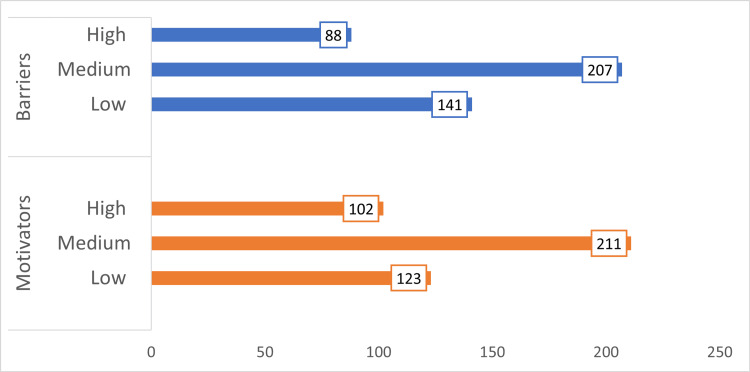
Categories of the total score on motivators and barriers in availing of preventive check-up The subsequent category data labels were given as frequency.

Figures 2, 3 show the various responses regarding motivators and barriers in availing of preventive health check-ups and Figure 4 shows the categories of the total score of motivators and barriers.

Table 2 shows the topmost scored barriers and motivators for a preventive health check-up. The mean ± SD of motivators and barriers score in availing of preventive health check-ups were 20.39 ± 2.73 and 21.23 ± 3.69, respectively.

**Table 2:** Topmost scored barriers and motivators

Barriers	Weightage score max (1308)
I have no awareness of the health check-up	1107
I am lazy to do a health check-up	1098
The procedure is uncomfortable for me	938
Motivators	
Insurance/work-related factors motivated me	1097
I have a health condition	933
I am doing it on the doctor's advice	933

The primary association of basic characteristics and motivators of preventive health check-ups showed that age, gender, education, occupation, marital status, presence of comorbidities, and vaccination status against COVID-19 were significantly associated with motivator score, whereas age, education, occupation, presence of comorbidities, and vaccination status were associated with barrier score. The age showed a significant negative correlation with the motivator/barrier score; as age increases, the motivator/barrier score decreases. Male gender, married subjects, subjects with higher education, stable occupation, with comorbidities, and COVID-19 vaccination had high motivator scores. Subjects with higher education, stable occupation, and COVID-19 vaccination had high barrier scores (Tables 3, 4).

**Table 3:** Linear regression analysis in predicting the motivators of preventive health check-ups Adjusted R^2^ = 58%, regression model fit, F value = 12.48, p-value < 0.001. * Significant p-value < 0.05; ^a^ Pearson correlation; ^b^ independent t-test; ^c^ one-way ANOVA test; ^#^ correlation coefficient.

			Motivator score	P-value obtained by primary analysis	B coefficient (95% CI), p-value
Age			-0.20^#^	<0.001^*,a^	-0.02 (-0.04 to -0.005), 0.01*
Gender		Female	20.06 (2.68)	0.01^*,b^	0.33 (-0.21 to 0.875), 0.11
		Male	20.73 (2.74)		
Marital status		Married	20.68 (2.72)	0.006^*,b^	-0.09 (-0.65 to 0.46), 0.30
		Unmarried and others	19.94		
Education		No formal education	19.31 (1.86)	<0.001^*,c^	1
		School education	20.44 (2.88)		0.19 (-0.62 to 1.01), 0.63
		Higher education	20.85 (2.84)		0.62 (-0.38 to 1.62), 0.22
Occupation		Unemployed	19.76 (2.44)	<0.001^*,c^	1
		Unstable income	20.7 (3.09)		0.56 (-0.31 to 1.42), 0.21
		Stable income	20.98 (2.77)		0.51 (-0.19 to 1.22), 0.15)
Presence of comorbidities		Yes	21.07 (2.86)	<0.001^*,b^	1.67 (1.14 to 2.23), <0.001*
		No	19.86 (2.49)		
Diagnosed with COVID-19	Yes		20.75 (2.68)	0.08^b^	
	No		20.25 (2.74)		
Vaccinated against COVID-19	Yes		20.78 (2.83)	<0.001^*,b^	-0.306 (-0.922 to 0.309), 0.09
	No		19.68 (2.38)		

**Table 4:** Linear regression analysis in predicting the barriers to availing of preventive health check-ups Adjusted R^2^ = 62%, regression model fit, F value = 11.68, p-value < 0.001. * Significant p-value < 0.05; a Pearson correlation; b independent t-test; c one-way ANOVA test; # correlation coefficient.

			Barrier score	P-value obtained by primary analysis	B coefficient (95% CI), p-value	
Age			-0.30^#^	<0.001^*,a^	-0.02 (-0.039 to 0.04), 0.25	
Gender		Female	21.08 (3.55)	0.39^b^		
		Male	21.39 (3.82)			
Marital status		Married	20.95 (3.63)	0.05^b^		
		Unmarried and others	21.67 (3.74)			
Education		No formal education	19.67 (2.52)	<0.001^*,c^	1	
		School education	20.02 (3.38)		0.07 (-0.96 to 1.10), 0.89	
					2.36 (1.08 to 3.65), <0.001*	
		Higher education	22.63 (3.78)			
Occupation		Unemployed	20.88 (3.35)	<0.001^*,c^	1	
		Unstable	10.63 (3.99)		-0.87 (-1.94 to 0.19), 0.11	
		Stable	22.11 (3.73)		-0.61 (-1.5 to 0.26), 0.17	
Presence of comorbidities		Yes	20.79 (3.62)	0.03^*,b^	0.118 (-0.57 to 0.81), 0.4	
		No	21.58 (3.70)			
Diagnosed with COVID-19	Yes		20.86 (3.67)	0.19^b^		
	No		21.38 (3.69)			
Vaccinated against COVID-19	Yes		21.92 (3.89)	<0.001^*,b^	-0.63 (-1.46 to 0.81)	
	No		19.98 (2.89)			

The linear regression analysis of significant variables with motivator score (Table 3) showed that age and the presence of co-morbidities were significantly associated with the motivator score after regressing with other variables. As one unit change occurs in age, the motivator score decreases by 0.02 and a one-unit change in the presence of comorbidities reduces the score by 1.68.

The linear regression analysis of significant variables with barrier scores showed that education was significantly associated with the motivator score after regressing with other variables. As one unit change occurs in education, it increases the barrier score by 1.37 (Table 4).

## Discussion

The study was conducted to estimate the proportion of people who have availed of preventive health check-ups and to determine the motivators and barriers to health check-ups among adults (age group = 18 years and above).

Out of 436 participants, only 130 (29.82%) had done preventive health check-ups in the past. A web report by a lab showed that 75% of Indians never get diagnostic testing done unless they are ill and their doctors order it [9]. In a Chinese survey, 48.94% of middle-aged and older Chinese people get preventative care examinations [10]. In contrast, a study done in Germany [11] and Vermont [12] showed that 53.4% and 81.1%, respectively, were reported participating in preventive health check-ups at least every two years. Such variation is due to regional variation and the individual's perception of health check-ups. In this study, subjects of young age, COVID-19 vaccinated, with stable occupations, and higher education availed preventive health check-ups.

A study done in Poland [13] shows that older age, having higher education, being occupationally active, having at least one chronic disease, and visiting a doctor in the past 12 months were significantly associated (p < 0.001) with a higher level of compliance with screening guidelines. A study done in Vermont among young adults showed that increased age and male gender showed to avail less frequent preventive health check-ups in correspondence to their counterparts [12]. A study done in Germany showed that people with an unfavourable risk factor profile, such as socioeconomically disadvantaged groups, smokers, physically inactive people, and those who eat a low amount of fruits and vegetables, are less likely to undergo health check-ups than those with a more favourable risk profile [14].

The study showed that non-awareness, idleness, and uncomfortable testing are the topmost barriers cited against doing preventive health check-ups and the motivators are insurance or work-related factors, doctor’s advice, and the presence of a health condition. Different studies have shown that lack of time, perception of being healthy, and constant contact with family physicians are the perceived hindrances in availing the health check-ups [15-17]. Also, few studies have shown low knowledge and attitudes such as unwillingness or care about the consequences, and low perceived severity of the health conditions were also barriers [18,19]. Studies have shown that the perception of security given by the health check-ups, understanding of the purpose of check-ups, and general obeyance towards doctor’s advice motivates to do the health check-ups [14,20-23].

In the study, younger age, male gender, married subjects, subjects with higher education, stable occupation, with comorbidities, and COVID-19 vaccinated had high motivator scores. Subjects with higher education, younger age, stable occupation, and COVID-19 vaccination had high barrier scores. A review has shown that lower education, younger age, reported smoking status, and living alone were the most apparent barriers to participating in a health check-up [6]. Age and education both had a substantial impact on the presence of a motivator or a barrier, with the increase or decrease of both acting as a double-edged sword. Younger age might make a person more proactive in checking disease status to prevent future ailments or not checking in the belief of being healthy. In theory, education enhances awareness, health literacy, communication, social inclusion, patience, self-confidence, self-efficacy, and motivation, which makes people more likely to seek preventative health care, as supported by numerous research [5,24-26]. This improved education operates as a barrier by making a person more confident in diagnosing or knowing about the ailment. The presence of a regular income and the requirement to undergo mandated preventative health examinations would have made stable occupations like salaried and businesspersons have a high motivating score. This stability can also act as a barrier where time constraints and social pressure prevents a person from being routine in the health check-up. Marriage would have made a person feel more pressurized to be at their optimum health and to prevent future risk, which made them avail the check-ups.

Male subjects showed significantly higher scores for motivators, which is in contrast to a study among health professionals where no difference based on gender in terms of barriers was noted [27]. Studies have shown females being highly motivated in doing a health check-up and masculinity leading to negative health promotion behaviours [6,28]. The subjects without comorbidities and who were COVID-19 vaccinated had higher motivation for a preventive health check-up. The subjects who were diagnosed and vaccinated against COVID-19 had a higher barrier in preventive health check-ups.

Strengths and recommendations

The study was first of its kind that assessed the proportion of subjects availing preventive health check-ups and its barriers/motivators. The substantial sample size and the analytical design, which allowed for potential confounder adjustments, are a few of the study's strengths. High data consistency was more likely because of the thorough questionnaire and a dependable survey-interview team were used.

Further studies should be done to analyse the satisfaction of subjects who availed of preventive health check-ups. Comprehensive qualitative research ought to be conducted to evaluate the underlying obstacles to receiving preventative health examinations.

Limitations

The measurements of barriers and motivators were self-reported and therefore subject to respondent bias. The study have been conducted after the period of the COVID-19 pandemic, which would have made many of them avail of few health check-ups or prevented them from doing it. The cross-sectional nature of the study prevented from assessing the beneficial effects of general health checks on morbidity, hospitalization, disability, worry, additional physician visits, or absence from work.

## Conclusions

In the study, only one-third of the respondents had previously received any form of preventive health check-up. Subjects who have availed of preventive health check-ups were young, COVID-19 vaccinated, and with a stable occupation and a higher degree of education. According to the survey, the top barriers against getting a preventative health check-up are non-awareness, idleness, and uncomfortable testing, while the motivators are insurance or work-related factors, doctor's advice, and the presence of a health ailment. Age, education, and comorbid status turned out to be predictors of motivation/barriers in availing of preventive health check-ups.
